# Investigating the Relationship Between CT-Derived Sarcopenia Index and Patient Acceptable Symptom State (PASS) in Psoriatic Arthritis: A Preliminary Analysis

**DOI:** 10.3390/jcm15062105

**Published:** 2026-03-10

**Authors:** Sibel Bakirci, Gulperi Ates Cetinkaya, Gokhan Sargin, Nazmi Kastan, Mustafa Sagan, Iclal Erdem Toslak

**Affiliations:** 1 Division of Rheumatology, Department of Internal Medicine, Antalya Training and Research Hospital, Antalya 07070, Turkey; 2 Department of Internal Medicine, Antalya Training and Research Hospital, Antalya 07070, Turkey; perigulates@gmail.com; 3 Division of Rheumatology, Department of Internal Medicine, Adnan Menderes University Hospital, Aydin 07070, Turkey; gokhan_sargin@hotmail.com; 4 Department of Radiology, Antalya Training and Research Hospital, Antalya 07070, Turkey; nazmikastan@gmail.com (N.K.);

**Keywords:** sarcopenia, psoriatic arthritis, biologic agents, computed tomography, PASS, patient acceptable symptom state

## Abstract

**Background:** This proof-of-concept study examined the association between CT-derived sarcopenia indices and achievement of Patient Acceptable Symptom State (PASS) in a metabolically homogeneous subgroup of patients with psoriatic arthritis (PsA) receiving biologic therapy. **Methods:** We retrospectively analyzed 26 PsA patients without diabetes, hypertension, or thyroid disorders who were treated with TNF or IL-17 inhibitors and underwent opportunistic CT imaging at the L2 vertebral level. Body composition parameters included total abdominal muscle area (TAMA), subcutaneous adipose tissue (SAT), and sarcopenia index (SI = TAMA/height^2^). PASS achievement at 12 weeks was assessed retrospectively. Receiver operating characteristic (ROC) analysis was performed. **Results:** Fourteen patients achieved PASS. Compared with non-achievers, these patients had significantly higher TAMA, SAT, and SI values (all *p* < 0.05). ROC analysis identified an SI threshold of 4657.5 cm^2^/m^2^ (sensitivity 78.6%, specificity 83.3%, *p* = 0.003). **Conclusions:** In this metabolically homogeneous PsA subgroup, higher CT-derived muscle mass measures were associated with PASS achievement at 12 weeks. These findings are hypothesis-generating and require validation in larger prospective cohorts.

## 1. Introduction

The progressive decline in skeletal muscle mass and its associated function, known as sarcopenia, is increasingly recognized in chronic inflammatory conditions such as psoriatic arthritis (PsA) [1,2]. While aging is a primary driver, secondary sarcopenia in PsA may arise from systemic inflammatory burden, metabolic disturbances, and reduced physical activity [3,4], contributing to diminished quality of life and functional independence [4].

According to international consensus criteria, sarcopenia encompasses not only reduced muscle mass but also muscle weakness and/or impaired physical performance. In this study, muscle mass was quantified using CT-derived body composition analysis; therefore, the sarcopenia index represents a muscle-mass-based metric rather than a comprehensive assessment of the sarcopenia syndrome. CT-derived quantification provides an objective and reproducible measure of skeletal muscle obtained from routine imaging; however, muscle mass alone is insufficient for formal sarcopenia diagnosis, and the relationship between CT-quantified muscle mass and functional outcomes in PsA remains incompletely understood [1,2,3,4].

The advent of biologic agents, specifically tumor necrosis factor inhibitors (TNFi) and interleukin-17 inhibitors (IL-17i), has transformed PsA treatment, although patient responses remain heterogeneous [5]. This variability highlights the need for reliable objective markers associated with patient-centered outcomes such as the Patient Acceptable Symptom State (PASS), which reflects whether patients consider their health state satisfactory [6,7].

Computed tomography (CT) enables precise quantification of body composition and allows opportunistic assessment without additional radiation exposure [8,9,10,11]. However, the role of CT-derived sarcopenia indices in predicting PASS achievement after biologic therapy in PsA remains unclear.

This proof-of-concept study examined the association between preserved muscle mass, quantified by CT-derived sarcopenia indices, and PASS achievement in a metabolically homogeneous subgroup of PsA patients receiving biologic therapy.

## 2. Methods

### 2.1. Patient Selection and Study Framework

This retrospective, single-center, exploratory study was approved by the Local Ethics Committee of Antalya Training and Research Hospital (No: 2024-031) and conducted in accordance with the Declaration of Helsinki. Because of the retrospective design and the use of de-identified data, informed consent was waived.

This proof-of-concept study applied a selective patient identification strategy to minimize the confounding effects of metabolic disease on muscle physiology and inflammatory responses. Consecutive PsA patients fulfilling CASPAR criteria who initiated TNFi or IL-17i therapy between 2024 and the study’s inception were screened. Baseline disease activity scores (e.g., DAPSA, MDA, CDAI) were not systematically available due to inconsistent documentation in electronic medical records.

Patients with documented diabetes mellitus (Type 1 or Type 2), hypertension requiring pharmacological treatment, or thyroid disorders (hypothyroidism, hyperthyroidism, or thyroiditis) were excluded. Of 102 screened patients, 26 met the inclusion criteria. The high exclusion rate (74.5%) reflects intentional selection of a metabolically homogeneous subgroup to isolate the independent contribution of muscle mass to treatment response. Metabolic comorbidities were identified through a detailed electronic record review. Hypertension was defined by prior diagnosis or antihypertensive use; diabetes by documented diagnosis, fasting glucose ≥ 126 mg/dL, HbA1c ≥ 6.5%, or antidiabetic therapy; and thyroid disorders by a history requiring hormone replacement or antithyroid treatment. These conditions are known to influence muscle metabolism and systemic inflammation and were therefore considered significant confounders.

Inclusion additionally required a thoracic CT scan extending to at least the L2 vertebral level within six months prior to biologic initiation for non-PsA indications. Patients receiving high-dose corticosteroids (>10 mg/day) or with poor-quality CT images were excluded, while standard background therapies were permitted. This approach allowed focused evaluation of the association between sarcopenia indices and PASS achievement in a metabolically selected PsA subgroup.

### 2.2. Evaluating Clinical Response: PASS

The primary outcome was achieving PASS at 12 weeks. PASS is a patient-reported outcome that identifies the symptom level patients consider acceptable [6]. It was assessed retrospectively at the 12-week follow-up using the standard validated PASS question: “Taking into account all the ways your disease affects you, if you were to remain in the next few months as you were in the last 3 months, would you consider your current state as acceptable?” (Yes/No) [6,7]. Patients who answered “Yes” were classified as achieving PASS, and those who answered “No” were not.

This comparative assessment approach asks patients to evaluate their recent disease state relative to treatment initiation rather than recall specific baseline symptoms, which is consistent with validated retrospective use of PASS in rheumatology research. The three-month interval represents a clinically meaningful period with expected changes in disease activity, supporting the reliability of this comparison.

### 2.3. CT Protocol and Body Composition Analysis

All thoracic CT scans were obtained for non-PsA-related clinical indications (e.g., pulmonary evaluation or investigation of other organ-specific pathology). This opportunistic imaging approach did not expose patients to additional ionizing radiation beyond standard medical care. Scans were performed on multidetector CT systems (Somatom Go.Up; Siemens Healthineers, Erlangen, Germany or Brilliance 64; Philips Healthcare, Best, The Nether-lands) with dose-modulation technology (120 kV, 74 mA with automatic exposure control; slice thickness 0.75–3 mm). Body composition analysis was conducted retrospectively on archived imaging data without additional radiation exposure using AiCRO software (version 1.0; Asan Medical Center, Seoul, Republic of Korea).

The L2 vertebral level was identified on axial CT images using standard anatomical landmarks, including vertebral morphology with bilateral transverse processes, sequential vertebral counting from L5 cranially, iliac crest positioning corresponding to the L4–L5 interspace, and visualization of the psoas muscles flanking the vertebral body. A single axial slice at L2 was selected and verified by an experienced radiologist (NK) prior to segmentation to ensure consistency.

All images were assessed for technical adequacy before analysis. Adequate image quality required clear visualization of the L2 vertebral level and anatomical landmarks, absence of significant motion artifact, sufficient field of view including bilateral psoas and abdominal musculature, and adequate contrast to distinguish skeletal muscle (Hounsfield Units −29 to +150) from adipose tissue. Images not meeting these criteria were excluded. Segmentation was performed semi-automatically using Asan J software (2018.2.19 Level 1 certification), with manual correction by the radiologist (NK), who was blinded to clinical outcomes.

**Figure 1:**
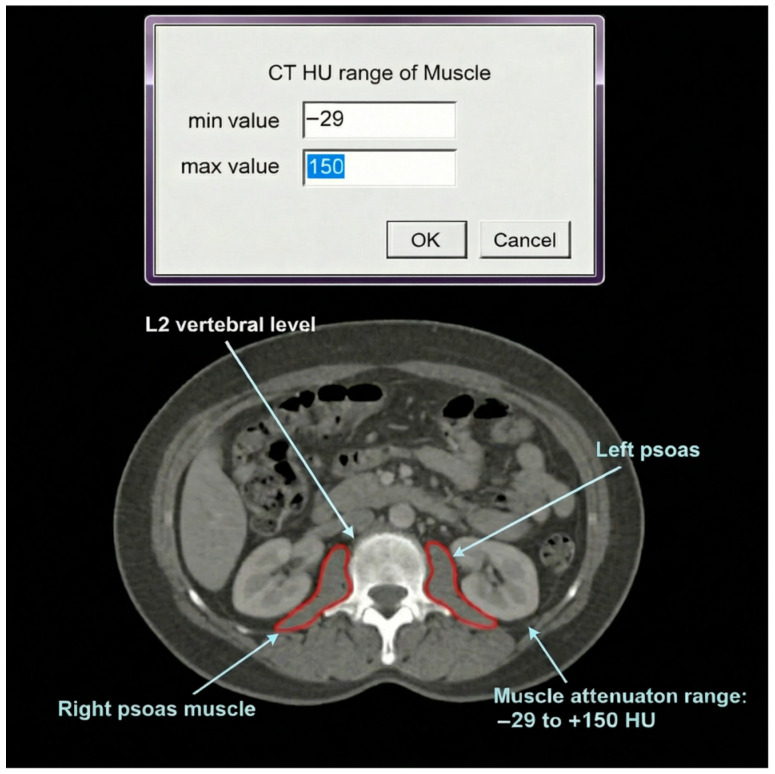
Axial CT image at the L2 vertebral level demonstrating the segmentation methodology for muscle quantification. Bilateral psoas muscles are outlined in red as a representative example of the semi-automated segmentation process. The muscle attenuation range was defined by Hounsfield Unit (HU) thresholds of −29 (minimum) to +150 (maximum), as shown in the input window at the top of the figure. Note: While this figure shows psoas muscle segmentation for illustrative purposes, the total abdominal muscle area (TAMA) used in all analyses included all skeletal muscle compartments at the L2 level (psoas, erector spinae, quadratus lumborum, rectus abdominis, oblique muscles, and transversus abdominis), not solely the psoas muscles depicted here.

Total abdominal muscle area (TAMA) was defined as the sum of all skeletal muscle compartments visible on the selected L2 slice, including psoas, erector spinae, quadratus lumborum, rectus abdominis, internal and external oblique, and transversus abdominis. The total cross-sectional area was calculated in cm^2^ using validated HU thresholds [9,10] (Figure 1).

### 2.4. Statistical Methodology

Data analysis was performed using IBM SPSS v25. Continuous variables were presented as mean ± SD or median [IQR], and categorical variables as frequencies. Normality was assessed using the Kolmogorov–Smirnov test. Group comparisons were conducted using independent *t*-tests or Mann–Whitney U tests for continuous variables, and chi-square or Fisher’s exact tests for categorical variables.

Optimal cutoff values for dichotomizing continuous body composition parameters (TAMA, SAT, and SI) were determined using the Youden Index (J = Sensitivity + Specificity − 1), defined as the maximum vertical distance between the ROC curve and the diagonal reference line. For each parameter, the cutoff maximizing the Youden Index was selected as the optimal threshold for predicting PASS achievement. Corresponding sensitivity and specificity values were calculated and reported.

Receiver Operating Characteristic (ROC) curve analysis was performed to evaluate the discriminative ability of body composition parameters in predicting PASS achievement. The area under the curve (AUC) quantifies the discriminative ability of each parameter, with values ranging from 0.5 (no discrimination) to 1.0 (perfect discrimination). Sensitivity and specificity values were calculated for each parameter at the identified cutoff values.

## 3. Results

### 3.1. Patient Selection and Characteristics

Out of 102 screened individuals, 26 met the strict inclusion criteria. Seventy-six patients (74.5%) were excluded for the following reasons: diabetes mellitus (*n* = 28), hypertension (*n* = 31), thyroid disorders (*n* = 12), and insufficient imaging data (*n* = 5). This selective approach enhances internal validity but limits generalizability to the broader PsA population. The cohort was predominantly female (88.5%), with a mean age of 50.7 ± 6.3 years and a mean disease duration of 9.4 ± 5.0 years. At week 12, 14 patients (53.8%) reported achieving a PASS, while 12 (46.2%) did not. Most patients had previously used csDMARDs, such as methotrexate (92%) or sulfasalazine (76%). Adalimumab was the most common biologic (84%), followed by secukinumab (48%).

### 3.2. Comparison of Body Composition Parameters

As detailed in Table 1, patients who achieved PASS exhibited significantly more robust muscle and adipose tissue profiles at baseline. The mean TAMA was significantly higher in the PASS group (126.42 ± 22.36 cm^2^) compared to those who did not achieve PASS (101.74 ± 40.79 cm^2^) (*p* = 0.002). Similarly, the median SI was markedly higher in patients reaching an acceptable symptom state [5601.9 (3102–14,320) cm^2^/m^2^] than in those who did not [3288.6 (1789–20,649) cm^2^/m^2^] (*p* = 0.003). Subcutaneous adipose tissue (SAT) area was also significantly greater in the PASS group (243.12 ± 134.39 cm^2^ vs. 150.86 ± 58.64 cm^2^, *p* = 0.03). However, visceral abdominal adipose tissue (VAT) area did not show a statistically significant difference between the two groups (132.19 ± 63.12 cm^2^ vs. 109.48 ± 52.85 cm^2^, *p* = 0.36).

### 3.3. Predictive Modeling and ROC Analysis

ROC curve analysis was performed to evaluate the discriminative ability of body composition parameters in predicting PASS achievement at week 12. The area under the curve (AUC) quantifies the discriminative ability of each parameter, with values ranging from 0.5 (no discrimination) to 1.0 (perfect discrimination). As shown in Table 2, the diagnostic performance of each body composition parameter is presented.

**Figure 2:**
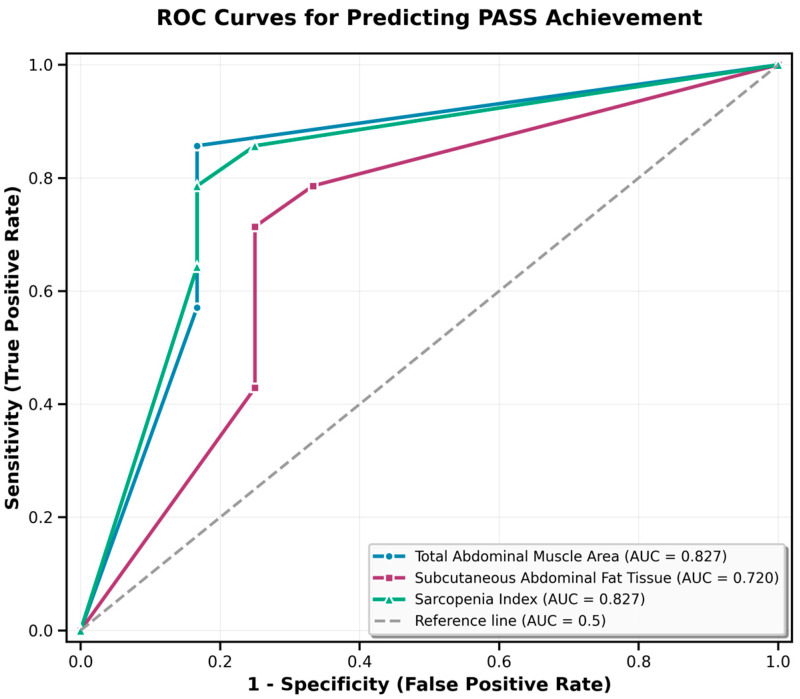
Receiver Operating Characteristic (ROC) curves for CT-derived body composition parameters in predicting PASS achievement in psoriatic arthritis patients treated with biologic therapy. The curves demonstrate the discriminative performance of total abdominal muscle area (TAMA, blue line), sarcopenia index (SI, green line), and subcutaneous adipose tissue area (SAT, red line). The area under the curve (AUC) for TAMA was 0.827 (95% CI: 0.658–0.996, *p* = 0.002), with an optimal cut off of 108.88 cm^2^ corresponding to 85.7% sensitivity and 83.3% specificity. The AUC for the sarcopenia index (SI) was 0.827 (95% CI: 0.665–0.989, *p* = 0.003), with an optimal cutoff of 4657.5 cm^2^/m^2^ corresponding to 78.6% sensitivity and 83.3% specificity. The AUC for subcutaneous adipose tissue (SAT) was 0.720 (95% CI: 0.523–0.917, *p* = 0.03), with an optimal cutoff of 161.61 cm^2^ corresponding to 71.4% sensitivity and 75.0% specificity.

The diagnostic performance of body composition parameters in predicting PASS achievement was evaluated using Receiver Operating Characteristic (ROC) curve analysis. The area under the curve (AUC) quantifies the discriminative ability of each parameter, with values ranging from 0.5 (no discrimination) to 1.0 (perfect discrimination). TAMA demonstrated an AUC of 0.827 (95% CI: 0.658–0.996, *p* = 0.002). Similarly, the sarcopenia index (SI) demonstrated an AUC of 0.827 (95% CI: 0.665–0.989, *p* = 0.003). Subcutaneous adipose tissue (SAT) demonstrated an AUC of 0.720 (95% CI: 0.523–0.917, *p* = 0.03). These exploratory findings suggest associations between CT-derived body composition measures and PASS achievement; however, these results require validation in prospective cohorts before clinical application (Figure 2).

## 4. Discussion

This exploratory proof-of-concept study suggests that baseline CT-derived body composition metrics may serve as biomarkers of PASS achievement in patients with PsA undergoing biologic therapy. ROC analysis identified optimal cutoff values for TAMA (108.88 cm^2^), SAT (161.61 cm^2^), and SI (4657.5 cm^2^/m^2^) that demonstrate reasonable discriminative ability for predicting PASS achievement. These findings support the notion that body composition metrics may be associated with therapeutic outcomes in chronic inflammatory diseases and warrant validation in larger prospective cohorts. Skeletal muscle health, as quantified by these imaging-derived metrics, may represent an important modifiable factor in optimizing treatment response in patients with inflammatory arthropathies [10,11].

The prevalence of sarcopenia in PsA has been reported to range from 9.1% to over 60%, depending on diagnostic criteria and patient population [12]. Takami et al. [13] demonstrated that PsA patients exhibit significantly lower lean body mass than healthy controls, likely mediated by pro-inflammatory cytokines such as TNF-α and IL-6. Our findings are consistent with Piętowska et al. [12], who suggested that biologic therapies may partially reverse inflammatory sarcopenia by restoring anabolic balance. Together, these data support the hypothesis that preserved muscle mass may be associated with improved patient-reported outcomes.

Subcutaneous adipose tissue (SAT) was also associated with PASS achievement in this exploratory analysis, consistent with systematic reviews evaluating body composition in spondyloarthritis [14]. However, the clinical and biological significance of this association remains unclear.

Using PASS as the primary outcome provides a patient-centered perspective. PASS identifies the symptom level that patients consider acceptable [6,7], and achieving PASS has been associated with lower disease impact on daily life [15]. By linking CT-derived body composition measures to this threshold, our study connects objective imaging metrics with subjective patient-reported outcomes.

Our study is among the first to evaluate CT-derived SI at the L2 vertebral level in relation to PASS achievement in PsA. Although L3 is more commonly used for body composition analysis [9,10], L2 and L3 measurements show high correlation for quantifying abdominal muscle and adipose tissue. Nevertheless, correlation does not establish equivalence for predicting clinical outcomes. The cutoff values derived from our L2-based analysis (SI > 4657.5 cm^2^/m^2^; TAMA > 108.9 cm^2^) may not be directly applicable to L3-based studies, and methodological heterogeneity may limit comparability across cohorts. Standardized imaging protocols are needed to improve reproducibility.

The statistical associations observed in this study should be interpreted in light of the modest sample size and exploratory design.

Several limitations warrant consideration. The retrospective design introduces potential recall bias in PASS assessment. Although patients were asked to compare their recent disease state with their baseline state, which may reduce recall error compared with absolute baseline recall, retrospective outcome measurement remains susceptible to bias. PASS has been validated in PsA in prospective and cross-sectional settings [16,17], but validation of retrospective PASS recall is lacking. Muscle segmentation was performed at the L2 vertebral level rather than the conventional L3 level. Although strong anatomical correlation between L2 and L3 has been reported [7,8], predictive equivalence for clinical outcomes cannot be assumed. Additionally, excluding patients with metabolic comorbidities resulted in a highly selected cohort. Hypertension, diabetes mellitus, and thyroid disorders are common in PsA [18], and 74.5% of screened patients were excluded for these reasons. While this strategy reduced confounding and allowed isolation of the relationship between muscle mass and PASS achievement, it limits generalizability to metabolically heterogeneous populations.

In conclusion, in this metabolically selected PsA subgroup, higher CT-derived muscle mass measures were associated with PASS achievement at 12 weeks. These findings are exploratory and require validation in prospective, representative cohorts.

## Figures and Tables

**Table 1:** Comparison of demographic and CT-derived body-composition parameters between patients who achieved PASS and those who did not.

Parameters	PASS Achieved (***n*** = 14)	PASS Not Achieved (***n*** = 12)	***p***-Value
Age (years), mean ± SD	49.2 ± 5.1	52.5 ± 7.3	0.20
Gender (Female/Male), n	12/2	11/1	1.00
Height (m), mean ± SD	1.61 ± 0.06	1.63 ± 0.08	0.52
Disease duration (years), mean ± SD	10.1 ± 5.1	8.6 ± 4.9	0.46
BMI (kg/m^2^), mean ± SD	32.5 ± 5.5	28.5 ± 7.9	0.15
TAMA (cm^2^), mean ± SD	126.42 ± 22.36	101.74 ± 40.79	0.002
VAT (cm^2^), mean ± SD	132.19 ± 63.12	109.48 ± 52.85	0.36
SAT (cm^2^), mean ± SD	243.12 ± 134.39	150.86 ± 58.64	0.03
SI (cm^2^/m^2^), median [25th–75th p]	5601.9 [3102–14,320]	3288.6 [1789–20,649]	0.003

Abbreviations: BMI = body-mass index.

**Table 2:** Receiver Operating Characteristic (ROC) Analysis: Diagnostic Performance of Body Composition Parameters in Predicting PASS Achievement.

Variable	Cutoff Value	AUC	95% CI	Sensitivity	Specificity
TAMA	108.88 cm^2^	0.827	0.658–0.996	78.6%	83.3%
SAT	161.61 cm^2^	0.720	0.523–0.917	71.4%	75.0%
SI	4657.5 cm^2^/m^2^	0.827	0.665–0.989	71.4%	83.3%

## Data Availability

Available upon reasonable request.
